# Ulcerated Cancer of Stomach

**Published:** 1879-11

**Authors:** W. L. Herriman

**Affiliations:** Port Hope, Ontario


### ARTICLE III.

ULCERATED CANCER OF STOMACH. I. History of case of Rev. Wm. McCullough, Gore's Landing, Ontario, by W. L. HERRIMAN, M.D., L.C.R.C.P.M.S., Port Hope, Ontario; II. Clinical record, by J. R. MCCULLOUGH, M.D., Chicago, Ills.; and, III. Autopsy record, by DRS. HERRIMAN, MCCULLOUGH and GEO, RIDDLE, of Cold Springs, Ont. Autopsy made September 21, 1879, by said gentlemen.

I. *History.*—The Rev. Wm. McCullough, sometime in February, 1879, received an injury by being thrown from his cutter. In March and the subsequent months he had pain in the stomach, chest and kidneys, constipation and occasional vomiting without nausea. A slimy or thin fluid would come into his mouth and then he would vomit; perhaps once a week or fortnight. He generally vomited food with "slime."

In the second week of June, he had occasion to drink freely of cold water, to allay a bad feeling in his stomach. He soon vomited and threw up what was black as tar, with a good deal of pain. Frequent vomitings continued without any assignable cause. The first ejected portion was often a little food, then came a great quantity of liquid, with dark material floating in it, watery and very gluey or ropy. He knows that he vomits a great deal more than he takes into the stomach ; often a week elapses between these attacks. He feels relieved after free vomiting, and is quite sure the ejected matter is not bile. The taste and smell of this are quite offensive. On last Tuesday (August 12) he had a chill and trembling, when he vomited freely, and then first saw a tinge of bile. On Friday, the 15th, he vomited again, but no bile ; it was a dark brown-colored mass. His wife said that it was very ropy. The patient did not feel relieved after this attack, as after others, but vomited again on Sunday, the 17th, and was much prostrated all day. He has had hiccough continuously for the last twenty-four hours, and more or less every day for several weeks, having taken no nourishment except twice a very little beef tea, since last Thursday (5 or six days). On Saturday he drank half a cup full at a time. On Tuesday, the 19th, he threw up some undigested pearl barley, which was taken in some chicken broth a week ago ; also a berry of the black currant, taken in the month of July. The food he vomits is generally unaltered. The stool of to-day (August 19) is reported as "black as tar," and of about the same consistency. He began to emaciate some months ago ; in May last noticed he had lost a great deal of flesh, and has continued to lose flesh and get weaker ever since. I think I can discover a small induration at the pyloric orifice of the stomach, the hardness being not very decided. It is either an abscess or a cancer ; also a rounded hardness, which I take to be in the region of the gall bladder. I can press the width of two fingers freely up under the right ribs, in the region of the liver, which causes pain. There is no enlargement of the liver. There is trouble at times to make water ; it causes him great pain ; sometimes the urine dribbles and sometimes flows freely. He does not sleep well, though there is no constant pain. Tongue slightly coated, a little red at tip.

Aug 25.—I visited him Saturday, the 23d, and still found him feeble, yet his pulse keeps as good as could be expected.

Hiccough, I think, not quite so bad ; still vomits, he says, everything. But I cannot believe that there has been much, if any, of the dark flocculi, as at first, or that he has passed any dark, tarry stools since the day I visited him, as reported to you (Aug 19). Some slight tendency to looseness in discharges, for which I ordered enemata of tr. opii and starch, omitting the pills. I continued the quinine and bismuth and morphine when required. I wrote Dr. Riddle to give some musk or carbolic acid, according to suggestion. I do not find in to-day's examination any particular indication of enlargement at the pylorus, as I wrote before that I thought I could detect some slight enlargement—possibly there is none. What I felt may have been from some other cause. He does not seem to suffer from any great pain. I tried to have more hope, but must say that the case as a whole has a very unsatisfactory aspect to me. I expect to see him on Wednesday (Aug. 27th, 1879) again.

W. L. HERRIMAN, M.D., L.C.R.C.P.M.S.
JOHN ST., PORT HOPE, Ontario.

2. *Record.*—Aug. 27th, 1879.—I arrived at the landing in answer to a summons, expecting to find Mr. McCullough dead. Found him resting and comparatively free from pain. He recognized me. There was slight uneasiness at the œsophageal orifice of the stomach. I found a tumefaction at lower curvature of the stomach and to the right of the median line, which was causing him much pain. I thought it to be within the transverse colon. He had been vomiting and had hiccough about every 12 hours, from 1 to 3, morning and afternoon. The looseness of bowels of last week ceased without the aid of the enema of tr. opii and starch as ordered by Dr. Herriman. Mrs. McCullough reported that the stools looked like matter and were very offensive. I sent a telegram to Dr. Herriman to meet me in consultation to-morrow.

Thursday, Aug. 28th.—Drs. Herriman, Riddle and myself consulted in regard to the case, and found decided enlargement at the pylorus. We concluded to change the treatment and give sodæ hyposulphite twice in 24 hours, to overcome the fermentation, alternated with salicylic acid twice in 24 hours; nourishment with enemata of beef-tea alternated with yolk of egg and milk beaten together, thus giving the stomach rest.

Friday, Sept. 5th.—He has passed the whole week without vomiting or hiccough, has slept much, suffered from occasional nausea, attended with much pain. We administered brandy and water freely, and kept his mouth cool with ice. He has been enabled to retain the beef-tea during the past week.

Saturday morning, Sept. 6th.—Hiccough returned about one o'clock this a. m. violently, succeeded by excessive vomiting of a dark ropy fluid. We discontinued enemata of nourishment in consequence of the pain caused in the region of tumefaction.

Tuesday, Sept. 9th.—Have ceased all treatment, administering only brandy and water. The vomiting continues to return about every 12 hours, lasting, off and on, for about 2 hours. The lower extremities are cold. He continues to micturate about every 5 or 6 hours. The urine is scant and very red. He is growing weaker with each vomiting attack.

Friday, Sept. 12.—Excessive hiccough and vomiting—considerable flapping of the tumefaction with every hiccough. Just preceding the vomiting, there has been audible a distinct sound in the stomach, as of a liquid pouring out from a vessel. Then there is a vacancy and stare in the eye—a death-like pallor of the countenance; pulse very low and thready, almost imperceptible; then an impulse of hiccough and then vomiting of pus. The dark-colored vomit has ceased. He passed to-day with vomit—a black currant. He bade us all farewell, and is evidently dying.

Monday, Sept. 15th.—He still lives, declines brandy, and only takes it when insisted upon. After vomiting, tongue swollen, mouth very sore, jaws so weak he has difficulty in swallowing. He lay all day in a stupor until 7 p. m., when the vomiting returned. The hands were constantly in motion over his chest and stomach. He has micturated scantily, twice during the day.

Friday night Sept. 19th.—At one this a. m. he had a severe and exhausting vomiting, the amount discharged being larger than on any previous occasion, and of the same purulent character. He rested during the forenoon and until 1 p. m., when the vomiting and hiccoughing returned. At 2 p. m. it ceased, but acute pain followed in the back, over the stomach and chest. About 3 p. m. he showed signs of nephritic pains, hands constantly over the bladder; had not micturated since 3 a. m. Wednesday morning. I inserted the catheter and drew off about 4 ounces of urine, with relief, and administered an hypodermic injection of morph. ($\frac{1}{8}$ grain) over the stomach. In a half hour he was quite relieved, and remained so until death occurred at 9:30 p. m. He had micturated involuntarily, very freely (judging from the appearance of the clothing.)

J. R. McCULLOUGH, M. D.

CHICAGO, Ills., U. S.

III.—*Record of Autopsy of the Body of Rev. William McCullough, Gores' Landing, Ont., Sep.* 20, 1879.

An opening into the abdominal cavity was made by an incision from the ensiform appendix of the sternum downwards to within two inches of the pubis, laterally in a line with the lower ribs about five inches each way. I found a scirrhous tumor or sac about the size of a goose's egg, suspended from the small curvature of the stomach, near to the pylorus, by adhesions. The tumor was vascular, thick and nobby, with the surface ruptured, and thick purulent pus exuding from it.

About two inches from the pyloric orifice we found several white nodules, from the size of a pea to that of an acorn.

The stomach, on its lower surface, exhibited extensive adhesions, also around its pyloric portion. It crowded into the left hypochondriac region quite to the left of the median line. The liver was crowded upward and contained white nodules resembling cicatrices.

Immediately behind the duodenum we found a large nodular mass, about the size of a cocoa-nut, with adhesions to the duodenum. The duodenum was extensively adherent to all the surrounding parts and totally obstructed. While prosecuting the dissection, the contents of the stomach became discharged, in consequence of ulceration of the coats. It was evident the fluids were retained during life by the extensive adhesions, and on account of these adhesions we found great difficulty in dissecting out the stomach. We removed the whole mass, consisting of the stomach, liver, pancreas, spleen and kidneys.

Upon opening the stomach, we found a number of seeds, some lemon, black currant and tomato; there was also in the stomach a large quantity of purulent matter; the inner surface was much congested.

About one-half an inch from the œsophageal orifice, was situated a perforating ulcer, the size of a silver dollar, having extensive adhesions to the diseased pancreas. The opening of this ulcer was filled with sloughs from the pancreas (in these sloughs were a number of the same kind of smaller seeds) into which could be traced a number of sinuses, leading to absesses in that organ.

The pyloric orifice was nearly surrounded by a corroding ulcer, occupying at least three-fourths of its circumference, and extending into the duodenum about half an inch.

At the entrance into the duodenum, were found three or four excrescences surrounding the orifice — and a multitude of the same kind of seeds.

The kidneys each contained cysts, each kidney having one cyst as large as the half of a hen's egg and formed immediately beneath the cortical covering and considerably at the expense of the kidney, being three-fourths of an inch deep and one inch long in its substance; the other cysts were smaller.

The gall-bladder was full of bile and much discolored.

The liver contained four small but deep seated abcesses upon its under surface, and six upon its upper surface, one of which upon being ruptured, discharged about two fluid drachms of viscid pus. These abscesses were the cicatrices spoken of before.

The bladder was not removed from its position, but an examination of it by the hand, indicated hardness with the same cystic feeling as the kidneys.

